# TRIM5α selectively binds a restriction-sensitive retroviral capsid

**DOI:** 10.1186/1742-4690-2-40

**Published:** 2005-06-20

**Authors:** Sarah Sebastian, Jeremy Luban

**Affiliations:** 1 Departments of Microbiology and Medicine, Columbia University, College of Physicians and Surgeons, 701 West 168^th ^Street, HHSC 1502, New York, New York 10032, USA

## Abstract

TRIM5 is a potent retrovirus inhibitor that targets viruses bearing particular capsid (CA) residues. In most primate species, retroviral restriction requires the C-terminal SPRY domain unique to the α-isoform of TRIM5, but the mechanism by which susceptible viruses are recognized and targeted for restriction is unknown. Here we show that TRIM5α binds retroviral CA from detergent-stripped virions in a SPRY-dependent manner with sufficient discrimination to account for the exquisite specificity of restriction.

## Findings

Two independent screens identified TRIM5 as a potent retrovirus restriction element that targets select viruses after entry into primate cells [1,2]. The biochemical basis for specificity of restriction is only evident in cells of the owl monkey where HIV-1 CA is recognized by the C-terminal cyclophilin domain that is unique to the TRIM5 orthologue found in this genus [2-4]. In all other primates, including humans and macaques, potent CA-specific restriction is conferred by the TRIM5α isoform [1,5-9], which possesses a C-terminal SPRY domain [10]. The mechanism by which TRIM5α selects retroviruses bearing particular CAs for restriction is unknown, though the TRIM5α SPRY domain is required for restriction and variation in SPRY amino acid residues determines the CA-specificity of given TRIM5α orthologues [9,11-13].

Conventional biochemical and two-hybid experiments failed to detect an interaction between TRIM5α and CA (SS and JL, unpublished data). The observation that non-infectious virus-like particles saturate TRIM5α-mediated restriction [14], but only if the particles bear a mature core from a restriction-sensitive virus [15,16] suggests that the TRIM5α SPRY domain recognizes a complex structure unique to the core of susceptible virions. Consistent with this model, expression within target cells of *gag*, *gag-pol*, or *gag *fragments encoding CA, CA-NC, or ubiquitin-CA-NC fusions, failed to block restriction activity (David Sayah and JL, unpublished data).

Retrovirion cores can be liberated from the viral membrane envelope by detergent [17]. HIV-1 virion cores were prepared with several different detergents and mixed with recombinant TRIM5 orthologues. After TRIM5 enrichment by affinity chromatography, CA associated with owl monkey TRIMCypA, as reported with other methods [3,4], but not with the equally potent HIV-1 restriction factor rhesus macaque TRIM5α (SS and JL, unpublished data).

We then selected murine leukemia virus (MLV) for study because, relative to HIV-1, MLV CA remains tightly associated with viral reverse transcription (RT) and preintegration complexes [18,19]. MLV strains bearing an arginine at CA residue 110 (so-called N-MLV) are highly susceptible to restriction by human TRIM5α whereas MLV virions bearing glutamate in this position (B-MLV) are completely resistant to restriction [5-8].

**Figure 1:**
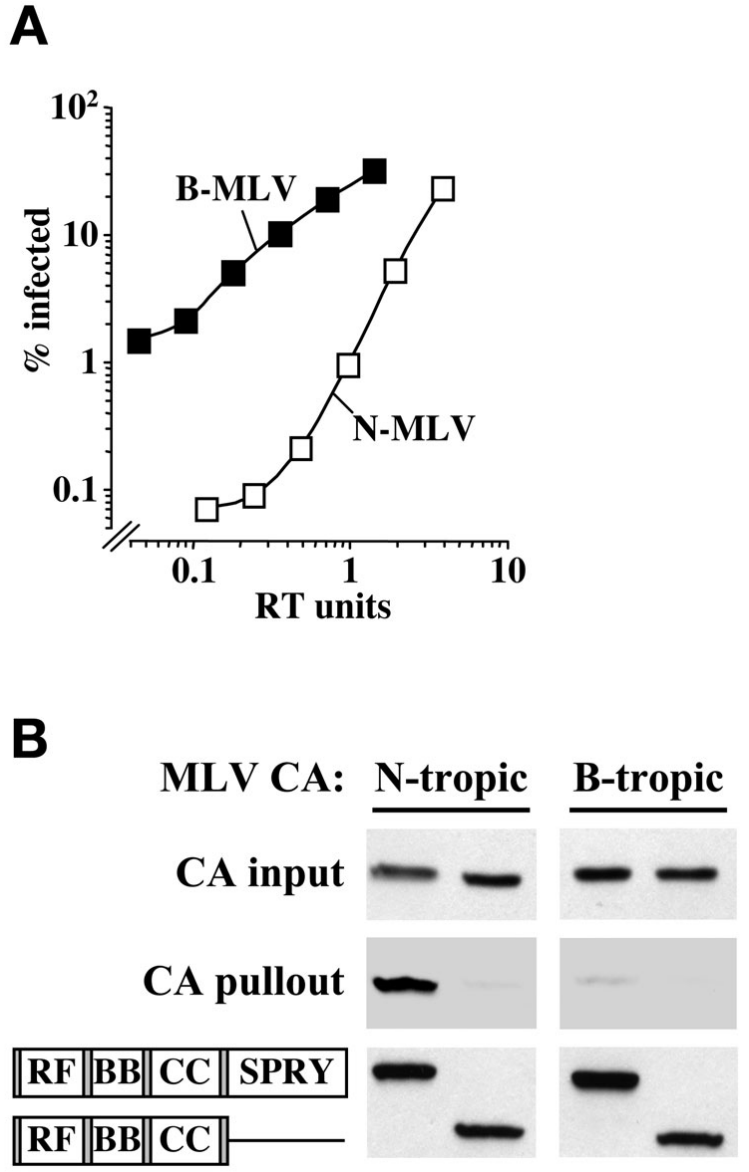
Human TRIM5α binds CA from restricted MLV virions. (A) HeLa cells were infected with VSV G-pseudotyped, N- and B-tropic MLV-GFP vectors after normalization for RT activity and infectivity on non-restrictive *Mus dunni *tail fibroblasts. The percentage of infected (GFP-positive) cells was determined by flow cytometry. (B) 293T cells were transfected with plasmids encoding glutathione S-transferase (GST) fusions with full-length TRIM5α or with TRIM5 lacking the SPRY domain. Cells were lysed (50 mM Tris pH 8.0, 150 mM NaCl, 1% NP-40, 0.1% SDS) and mixed for 2 hrs at 4°C with virions (N-MLV or B-MLV) that had been concentrated by acceleration through 25% sucrose. GST fusions and associated proteins were enriched on glutathione-sepharose beads and immunoblotted with goat anti-MLV CA antibody (CA pull-out), or anti-GST antibody (bottom panel). Unbound CA remaining in the binding reaction was probed with anti-MLV CA antibody (CA input). TRIM5 protein domains fused to GST are indicated schematically on the bottom left: RF, ring finger; BB, B box; CC, coiled-coil.

VSV G-pseudotyped N- and B-tropic MLV virions were produced as previously described [20] and, after normalization on non-restrictive *Mus dunni *cells, N-MLV was roughly 100-fold less infectious than B-MLV on HeLa cells (Figure 1A). Full-length human TRIM5α was then produced as a GST-fusion protein in 293T cells and mixed with purified N-MLV virions. CAp30, the major MLV core protein constituent, associated with TRIM5α (Figure 1B). CAp30 from B-MLV virions did not associate with TRIM5α (Figure 1B) demonstrating that TRIM5α binding was specific for restriction-sensitive CA. CAp30 did not associate with TRIM5 lacking the SPRY domain (Figure 1B), indicating that the SPRY-domain is required for CA-recognition.

Retroviral restriction specificity thus seems to be determined by TRIM5α binding to CA in a process that requires the SPRY domain. The fact that TRIM5α recognized retroviral CA presented by detergent-stripped virion cores, but not free CA protein, suggests that the SPRY domain recognizes a complex surface of multimerized CA. Once cores of restriction-sensitive viruses are singled out by the SPRY domain, TRIM5α blocks retroviral RT [1] by a mechanism that awaits elucidation. Our findings bring us one step closer to understanding how the potent antiviral activity of TRIM5α might be harnessed to block HIV-1 infection in people.

## List of abbreviations

HIV-1, human immunodeficiency virus; MLV, murine leukemia virus; TRIM, tripartite motif protein; RT, reverse transcriptase; CA, retroviral capsid protein; GST, glutathione S-transferase; RF, ring finger domain; BB, B box domain; CC, coiled-coil domain.

## Competing interests

The author(s) declare that they have no competing interests.

## Authors' contributions

SS and JL conceived the experiments and wrote the manuscript. SS performed the laboratory work. Both authors read and approved the final manuscript.

## References

[B1] Stremlau M, Owens CM, Perron MJ, Kiessling M, Autissier P, Sodroski J (2004). The cytoplasmic body component TRIM5alpha restricts HIV-1 infection in Old World monkeys. Nature.

[B2] Sayah DM, Sokolskaja E, Berthoux L, Luban J (2004). Cyclophilin A retrotransposition into TRIM5 explains owl monkey resistance to HIV-1. Nature.

[B3] Nisole S, Lynch C, Stoye JP, Yap MW (2004). A Trim5-cyclophilin A fusion protein found in owl monkey kidney cells can restrict HIV-1. Proc Natl Acad Sci U S A.

[B4] Berthoux L, Sebastian S, Sayah DM, Luban J (2005). Disruption of human TRIM5alpha antiviral activity by nonhuman primate orthologues. J Virol.

[B5] Keckesova Z, Ylinen LM, Towers GJ (2004). The human and African green monkey TRIM5alpha genes encode Ref1 and Lv1 retroviral restriction factor activities. Proc Natl Acad Sci U S A.

[B6] Hatziioannou T, Perez-Caballero D, Yang A, Cowan S, Bieniasz PD (2004). Retrovirus resistance factors Ref1 and Lv1 are species-specific variants of TRIM5alpha. Proc Natl Acad Sci U S A.

[B7] Yap MW, Nisole S, Lynch C, Stoye JP (2004). Trim5alpha protein restricts both HIV-1 and murine leukemia virus. Proc Natl Acad Sci U S A.

[B8] Perron MJ, Stremlau M, Song B, Ulm W, Mulligan RC, Sodroski J (2004). TRIM5alpha mediates the postentry block to N-tropic murine leukemia viruses in human cells. Proc Natl Acad Sci U S A.

[B9] Song B, Javanbakht H, Perron M, Park do H, Stremlau M, Sodroski J (2005). Retrovirus restriction by TRIM5alpha variants from old world and new world primates. J Virol.

[B10] Ponting C, Schultz J, Bork P (1997). SPRY domains in ryanodine receptors (Ca(2+)-release channels). Trends Biochem Sci.

[B11] Yap MW, Nisole S, Stoye JP (2005). A single amino acid change in the SPRY domain of human Trim5alpha leads to HIV-1 restriction. Curr Biol.

[B12] Sawyer SL, Wu LI, Emerman M, Malik HS (2005). Positive selection of primate TRIM5alpha identifies a critical species-specific retroviral restriction domain. Proc Natl Acad Sci U S A.

[B13] Stremlau M, Perron M, Welikala S, Sodroski J (2005). Species-Specific Variation in the B30.2(SPRY) Domain of TRIM5{alpha} Determines the Potency of Human Immunodeficiency Virus Restriction. J Virol.

[B14] Towers G, Collins M, Takeuchi Y (2002). Abrogation of Ref1 retrovirus restriction in human cells. J Virol.

[B15] Cowan S, Hatziioannou T, Cunningham T, Muesing MA, Gottlinger HG, Bieniasz PD (2002). Cellular inhibitors with Fv1-like activity restrict human and simian immunodeficiency virus tropism. Proc Natl Acad Sci U S A.

[B16] Towers GJ, Hatziioannou T, Cowan S, Goff SP, Luban J, Bieniasz PD (2003). Cyclophilin A modulates the sensitivity of HIV-1 to host restriction factors. Nat Med.

[B17] Welker R, Hohenberg H, Tessmer U, Huckhagel C, Krausslich HG (2000). Biochemical and structural analysis of isolated mature cores of human immunodeficiency virus type 1. J Virol.

[B18] Bowerman B, Brown PO, Bishop JM, Varmus HE (1989). A nucleoprotein complex mediates the integration of retroviral DNA. Genes Dev.

[B19] Fassati A, Goff SP (1999). Characterization of intracellular reverse transcription complexes of Moloney murine leukemia virus. J Virol.

[B20] Towers G, Bock M, Martin S, Takeuchi Y, Stoye JP, Danos O (2000). A conserved mechanism of retrovirus restriction in mammals. Proc Natl Acad Sci U S A.

